# Triple negative breast cancer and platinum-based systemic treatment: a meta-analysis and systematic review

**DOI:** 10.1186/s12885-019-6253-5

**Published:** 2019-11-08

**Authors:** Jessa Gilda P. Pandy, Joanmarie C. Balolong-Garcia, Mel Valerie B. Cruz-Ordinario, Frances Victoria F. Que

**Affiliations:** Section of Medical Oncology, Cancer Institute, St. Luke’s Medical Center, 279 E Rodriguez Sr. Ave, 1112 Quezon City, Metro Manila Philippines

**Keywords:** Triple negative breast cancer, Platinum chemotherapy

## Abstract

**Background:**

Triple negative breast cancer (TNBC) represents 15–20% of breast cancers. Due to its heterogeneity and high rates of relapse, there is a need to optimize treatment efficacy. Platinum chemotherapy is still controversial and currently not recommended as first-line treatment for TNBC. Recent studies have shown promising activity of this regimen. This study was done to evaluate the effect of platinum chemotherapy on pathologic complete response (pCR) after neoadjuvant treatment for early TNBC and progression-free survival (PFS) in metastatic TNBC.

**Methods:**

A systematic search of Pubmed, Embase, Cochrane, Clinical trials databases and hand search were done to identify randomized controlled trials (RCTs) investigating the use of platinum-based chemotherapy in adults with TNBC. Studies were appraised using the Cochrane Collaboration tool. Using the random effects model, pooled Odds ratios (ORs) with 95% confidence intervals (CI) for pCR, and Hazard Ratios (HRs) with 95%CI for PFS were analyzed.

**Results:**

Eleven RCTs were included (*N* = 2946). Platinum-based chemotherapy showed pCR benefit of 40%vs27% (OR1.75,95% CI 1.46–2.62,*p* < 0.0001) in the neo-adjuvant setting. Subgroup analysis showed increased pCR rates (44.6%vs27.8%) with platinum plus taxane regimen (p < 0.0001). In metastatic TNBC, three RCTs were analyzed (*N* = 531), platinum treatment did not show PFS advantage (HR1.16,95%CI 0.90–1.49,*p* = 0.24).

**Conclusion:**

Platinum chemotherapy is associated with increased pCR rates in TNBC, hence it is a viable option for patients in the neoadjuvant setting. Subgroup analysis showed that the combination of platinum and taxanes (Carboplatin/Paclitaxel) improved pCR. However, no PFS advantage was seen in metastatic TNBC. Given the current conflicting data in metastatic TNBC, further exploration with additional powered studies is needed.

## Background

Breast cancer is the most frequent malignancy among women worldwide. Approximately 10–20% of breast cancer cases is defined by the lack of expression of targetable biomarkers such as hormone receptors and human epidermal growth factor receptor 2 (Her2/neu) [1]. This subset of breast cancer is known as triple-negative breast cancer (TNBC). TNBC is one of the most aggressive subtypes of breast cancer, posing a treatment challenge. It is usually associated with larger tumor size, higher grade, and frequent nodal involvement [2]. Due to these characteristics, as many as 50% of patients diagnosed with early-stage triple-negative breast cancer experience disease recurrence, and 37% die in the first 5 years after surgery [3].

Whether in the early or advanced stages, chemotherapy represents the most widely accepted treatment for TNBC. The benefit of neo-adjuvant chemotherapy among TNBC has been evaluated in several trials. The GeparSixto trial in 2012 showed that neoadjuvant chemotherapy, using carboplatin, among TNBC and Her2-positive cases resulted in higher rates of pCR favoring TNBC (53% vs 33%) [4]. TNBC who attain pCR have improved event-free survival and overall survival, based on the CTNeoBC study in 2014, such that pCR may be used to convey prognostic information among this subset of patients [5].

In the most recent National Comprehensive Cancer Network (NCCN) guidelines [6], TNBC are treated with a combination of taxane- and anthracycline-based regimens. The benefit of taxane and anthracycline combination was compared to non-anthracycline regimen in the ABC Trials in 2017 which showed that in TNBC (31% of total patients) the hazard ratio (1.42 95% CI, 0.97 to 1.49) was in favor of the anthracycline regimen [7]. The current guidelines endorse the same protocol, however, due to the heterogeneity and high rates of relapse of TNBC, there is a need to optimize treatment efficacy, and develop novel chemotherapeutic regimens that can potentially improve survival outcomes [8].

TNBC commonly harbors BRCA gene mutations that make them especially susceptible to to DNA-damaging compounds such as platinum drugs [9]. Several neo-adjuvant clinical trials have evaluated the impact of adding platinum to standard chemotherapy. An early phase 2 trial by Silver et al. in 2010 [10], showed a pCR rate of 22% among all TNBC patients given neoadjuvant cisplatin. The GeparSixto trial by von Minckwitz et al. in 2014 [4], which included stage II or III TNBC (*n* = 588), demonstrated significant improvement in pCR with carboplatin (*p* = 0.005). However, the toxicities in the carboplatin arm caused a significantly higher rate of treatment discontinuation compared to the no carboplatin arm. In the CALGB 40603 trial by Sikov et al. in 2015, early TNBC patients showed higher pCR rates with the addition of carboplatin to the chemotherapy (*p* = 0.0089) [11]. To date, more recent trials have also investigated the role of platinum agents in TNBC, however the results are conflicting and studies are not powered enough to show statistically significant difference due to small populations [12].

Platinum chemotherapy has also been evaluated among TNBC in the metastatic setting. Currently, the 4th ESMO guidelines [13] recommend anthracycline-taxane chemotherapy as first line for treatment of advanced TNBC, while carboplatin may be considered for BRCA positive TNBC as second line treatment [14]. Existing studies have shown conflicting results for the use of platinum agents as first-line treatment for metastatic TNBC. In a retrospective cohort study by Zhang et al. in 2015 [15], longer PFS was observed in metastatic TNBC patients receiving platinum-based chemotherapy compared with nonplatinum-based therapy in the first line metastatic setting (7.8 months vs. 4.9 months, *p* < 0.001). Carey et al. in 2012 [16], showed in a randomized trial with metastatic TNBC patients, that combination cetuximab and carboplatin produced response rates in only less than 20% of patients. On the other hand, the CBCSG006 trial in 2015 by Hu et al. [17], showed increased PFS in patients with metastatic TNBC receiving cisplatin plus gemcitabine compared to patients receiving paclitaxel plus gemcitabine (7.73 vs 6.47 months, 95% CI 6·16–9·30). This suggests that platinum chemotherapy could be an alternative first-line chemotherapy in metastatic TNBC.

With the results of existing studies using platinum-based chemotherapy for TNBC, we hypothesize that this treatment can be considered as a potential component of both the neoadjuvant chemotherapy among early TNBC patients and as first-line chemotherapy for metastatic TNBC patients. Current breast cancer guidelines [6] do not have a firm recommendation regarding use of platinum in either setting outside a clinical trial setting. This present study has been conducted to provide up-to-date evidence on this topic and to provide a pooled analysis of existing results in order to further clarify the role of platinum-based chemotherapy in early and metastatic TNBC patients. The results of this study are deemed to aid in recommending platinum-based chemotherapy for TNBC patients.

## Methods

### Literature search strategy and study identification

**Fig. 1:**
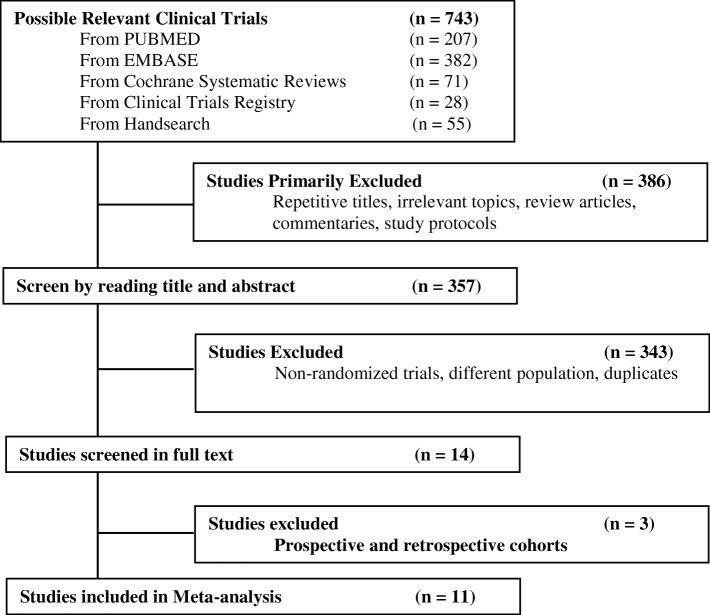
Flow-chart of the literature search

Eligible studies were identified by a systematic literature search of Pubmed, Embase, and Cochrane databases and the clinical trials registry using the date limits January 2006 and July 2018 (Fig. 1). A thorough hand-search through the references of selected studies was also conducted for any additional relevant studies. There were no language restrictions. The keywords used in the search strategy were ‘triple-negative’, ‘breast cancer’, ‘platinum’ and ‘chemotherapy’. Specific keywords and free text terms were combined with Boolean operators. The abstracts of the resulting studies were reviewed and full-text manuscripts were retrieved.

Resulting studies were selected and their eligibility was confirmed by three independent investigators. The systematic literature search was carried out independently by two authors (JP and MO) and any discrepancies were solved by discussion with a third author (JB).

### Selection criteria

Eligible studies had to satisfy the following inclusion criteria: [1] randomized controlled trials (RCTs), [9] Adult (18 years and above) TNBC patients, [3] treatment with platinum-based neoadjuvant chemotherapy in the experimental arm and platinum-free neoadjuvant chemotherapy in the control arm, [8] had pCR or PFS as outcomes. Studies excluded were those with [1] incomplete data on treatment and ER/PR/Her2 status, [9] non-RCTs, [3] RCTs involving other breast cancer subtypes, [8] and ongoing studies.

### Data extraction

The following information was extracted from each study: authors’ names, year of publication, study type, the total number of patients and chemotherapy regimens, type and dose of chemotherapy given, number of patients with pCR (defined as no residual invasive tumor in both the breast and the axilla, i.e. ypT0/is pN0) and PFS (defined as progression of disease from time of randomization) in the platinum-based and platinum-free chemotherapy arm.

### Quality evaluation

The collated evidence was evaluated using the Cochrane Collaboration tool [18]. Accordingly, the quality of each study was graded as A, B or C.

### Statistical analysis

Meta-analysis was conducted using Review Manager software (RevMan, version 5.3 for Windows; Cochrane Collaboration, Oxford, UK). The odds ratio (OR) and 95% confidence interval (95% CI) were calculated for the effect on pCR. Hazard ratios (HRs) and 95% CI were calculated for the effect of platinum-based versus platinum-free neoadjuvant chemotherapy in terms of PFS. A χ2 test was used to evaluate heterogeneity in the data. The random-effects model was used. To obtain a quantitative measure of the degree of inconsistency in the results of the studies, the Higgins I^2^ index was computed. I^2^ values 0–40% mean mild or non-significant heterogeneity; 30–60% may represent moderate heterogeneity; 50–90% represent substantial heterogeneity; and 90–100% represent considerable heterogeneity. Funnel plots were generated using RevMan to detect publication bias.

### Study objectives

The primary objective of this study is to compare the efficacy of platinum-based versus platinum-free chemotherapy in TNBC, specifically pCR among those who received neoadjuvant chemotherapy and PFS among those who received chemotherapy in the metastatic setting.

## Results

### Study selection and characteristics

A total of 743 articles were first identified for evaluation. Based on the inclusion and exclusion criteria described, 11 articles with 2946 patients were eligible for the meta-analysis. The search process is described in Fig. 1. Table 1 shows the characteristics of the included studies.

**Table 1:** Characteristics of eligible studies

Study	Year	Type	Population	Platinum-based regimen	Nonplatinum regimen	Outcomes
GEICAM/2006–03 [19]	2012	Randomized phase 2 trial	TNBC	EC × 4 ➔ DCb × 4 cycles	EC × 4 ➔ D × 4 cycles	pCR: EC-D 35% EC-DCb 45% (p = 0.606)
Ando [20]	2014	Randomized phase 2 trial	TNBC	CP q wk. x 12 ➔ CEF × 4 cycles	P q wk. × 12 ➔ CEF × 4 cycles	pCR: CP-CEF 31.8% P-CEF 17.6% (*p* = 0.01)
WSG-ADAPT-TN [21]	2017	Randomized phase 2 trial	TNBC	Nab-P/ Cb × 4	Nab-P/ Gem × 4	pCR Nab-P-Cb 45.9% Nab-P-Gem 28.7% *p* = 0.002
BrighT Ness [22]	2018	Randomized phase 3 trial	TNBC	Arm 1: PCb + Veliparib × 4 cycles Arm 2: PCb + Veliparib placebo × 4 cycles	Arm 3: Paclitaxel + Carboplatin placebo + Veliparib placebo × 4 cycles	pCR Arm 1 53% Arm 2 58% Arm 3 31%
CALGB 40603 [11]	2015	Randomized phase 2 trial	TNBC	Arm 3: P x 12w + Cb × 4 ➔ ddAC × 4 Arm 4: P × 12w + Cb × 4 + Bev × 9 ➔ ddAC × 4	Arm 1: P x 12w ➔ ddAC × 4 Arm 2: P × 12w + Bev q2w x 9w ➔ ddAC × 4	pCR (+) Cb 60% (−) Cb 46% (+) Bev 59% (−) Bev 48%
TNT Trial [23]	2018	Randomized phase 3 trial	TNBC	Cb	D	pCR Cb 6.7% D 3.3%
Gepar Sixto [4]	2014	Randomized phase 2 trial	TNBC	Cb + PDB x 18w or Cb + PDH x 18w	PDB x 18w or PDH x 18w	pCR (+) Cb 43.7% (−) Cb 36.9%
Zhang [24]	2016	Randomized phase 2 trial	TNBC	PCb	EP	pCR PCb 38.6% EP 14.0%
CBCSG006 [17]	2015	Randomized phase 3 trial	Metastatic TNBC	Gemcitabine/ Cisplatin × 8 cycles	Gemcitabine / Paclitaxel × 8 cycles	OS Gem/Cis × 8 59% Gem/P × 8 58% p = 0.611
Carey [16]	2012	Randomized phase 2 trial	Metastatic TNBC	Cet/ Cb	Cet	PFS: Cet + Cb 77% Cb 97% OS: Cet + Cb 83% Cb 83%
Fan [25]	2012	Randomized phase 2 trial	Metastatic TNBC	DP × 6 cycles	DX × 6 cycles	PFS: TP 25% TX 10% p < 0.001 OS TP 28% TX 10% p = 0.02

[*pCR* = pathological complete response; *DFS* = Disease free survival; *CRR* = Complete response rate; *OS* = Overall survival; *RR* = Response rate; *E* = Epirubin; *C/Cb* = Carboplatin; *D* = Docetaxel; *CEF* = Cyclophosphamide/Epirubicin/5-Fluorouracil; *P* = Paclitaxel; *Cet* = Cetuximab; *DP* = Docetaxel/Cisplatin *DX* = Docetaxel/Capecitabine; *Nab-P* = Nab-Paclitaxel; *Gem* = Gemcitabine; *ddAC* Doxorubicin/Cyclphosphamide; *Bev* = Bevacizumab; *H* = Trastuzumab]

Eleven randomized controlled trials were included in this study (*N* = 2946). Eight studies (*N* = 2415) which administered platinum-based neoadjuvant treatment were included for analysis of pCR and three studies (*N* = 531) which included metastatic TNBC were analyzed for PFS.

### Risk of bias assessment

Risk of bias assessment is summarized in Table 2. Using the Cochrane risk of bias assessment tool^22^, one study by Loibl et al. was graded A, while all the others were graded B primarily due to the lack of patient and staff blinding in these studies.

**Table 2:** Risk of Bias Summary using the Cochrane Collaboration’s Tool

Study	Selection	Performance	Exclusion	Detection	Quality
Alba 2012	B	B	B	B	B
Ando 2014	B	B	A	B	B
Carey 2012	B	B	A	B	B
Fan 2012	B	B	A	B	B
Gluz 2017	B	B	B	B	B
Hu 2015	A	B	A	B	B
Loibl 2018	A	A	A	A	A
Sikov 2015	B	B	B	B	B
Tutt 2018	A	B	A	B	B
Von Minkwitz 2014	B	B	A	B	B
Zhang 2016	B	B	A	B	B

#### pCR rates of TNBC patients treated with neoadjuvant platinum- versus non-platinum-based regimen

**Fig. 2:**
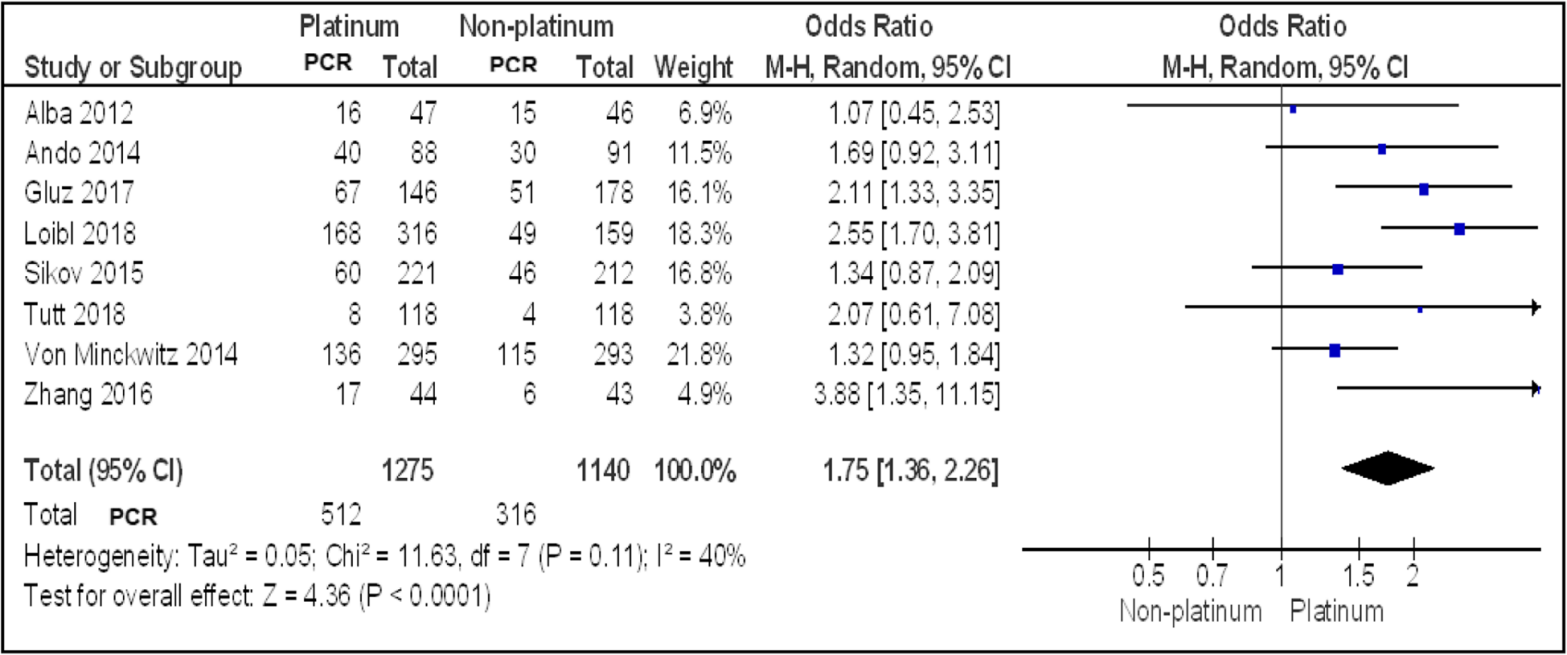
Forest plot showing pooled incidence of pCR in platinum vs non-platinum chemotherapy in early TNBC patients using random effects model with 95% confidence interval

Eight studies (*N* = 2415) reported pCR rates in TNBC patients. Figure 2 showed a statistically significant improved pCR rate (*P* < 0.0001) among patients treated with a platinum-based regimen than among those treated with a non-platinum-based regimen (40.1% vs 27.7%; OR, 1.75; 95% CI, 1.36–2.26). Trials have moderate heterogeneity (I^2^ = 40%) and evaluation with random effects model was done. The funnel plot (Fig. 5) generated showed mild asymmetry.

In all eight studies, Carboplatin was added as the platinum agent to an anthracycline- and taxane-based neoadjuvant chemotherapy. The study by Loibl^16^ used Paclitaxel with Veliparib, a PARP inhibitor, with or without Carboplatin while the studies by Sikov^14^ and Von Minckwitz^9^, both phase II RCTs added an anti-VEGF, Bevacizumab, to the chemotherapy regimen.

#### Subgroup analysis of pCR rates among TNBC patients treated with neoadjuvant platinum-based regimen

Subgroup analysis was done to remove heterogeneity among the trials, which may be attributed to the type of chemotherapy agent combined with platinum. Two subgroups were analyzed: platinum + taxane regimen and platinum + anthracycline regimen.

**Fig. 3:**
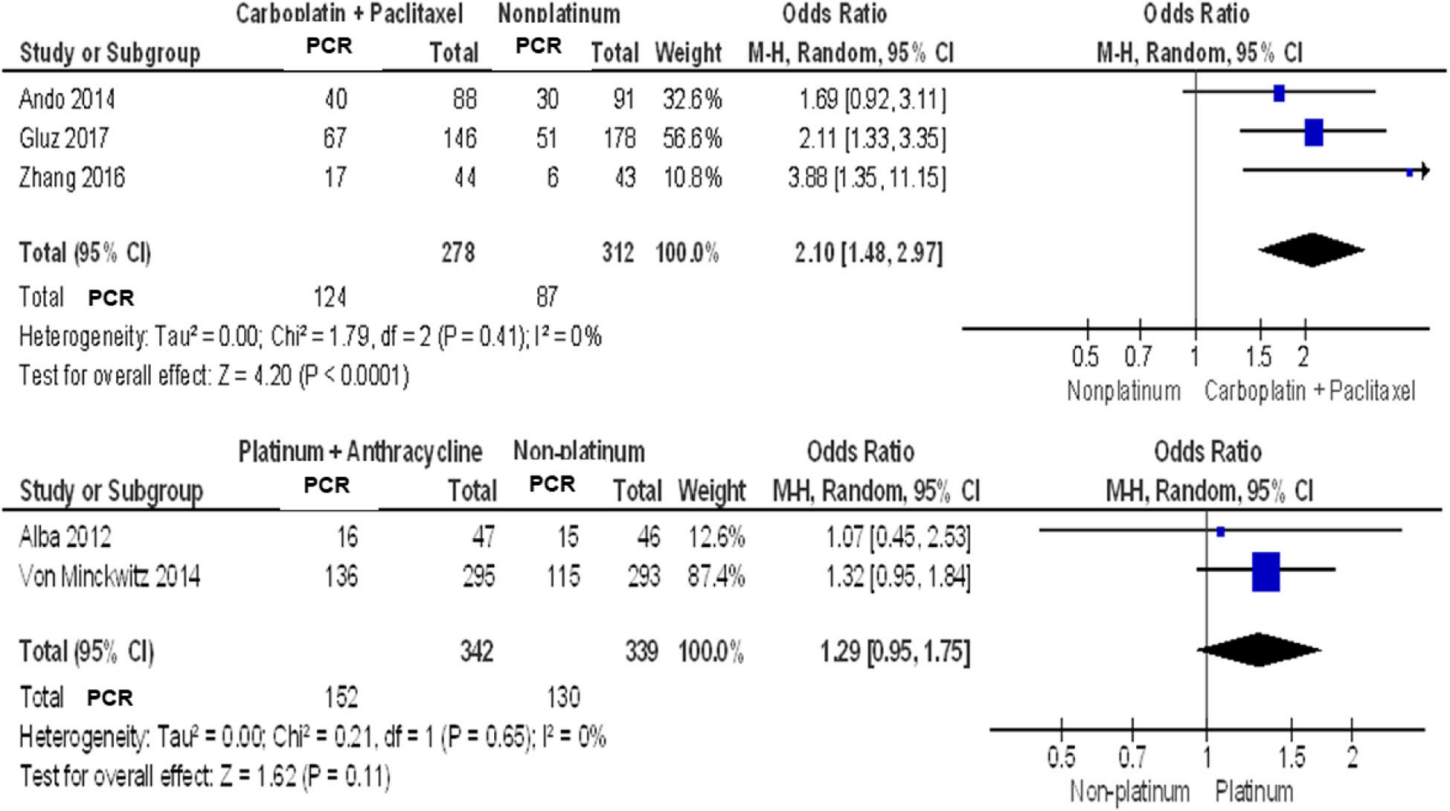
Forest plot showing pooled incidence of pCR in subgroup of TNBC patients given Carboplatin/Paclitaxel (Top), and Platinum/Anthracycline (Bottom)

Three [3] studies have platinum + taxane regimen (*N* = 590). As seen in Fig. 3, the pooled analysis showed statistically significant increase in pCR rates (44.6% vs 27.8%) among TNBC patients treated with a taxane (Paclitaxel) plus a platinum agent (Carboplatin) using random effects model (*p* value< 0.0001). With an I^2^ of 0, results of the three studies were homogenous. The second subgroup with two studies with platinum and anthracycline regimen, did not show any significant benefit.

#### PFS rates among metastatic TNBC patients treated with a platinum- or a non-platinum-based regimen

**Fig. 4:**
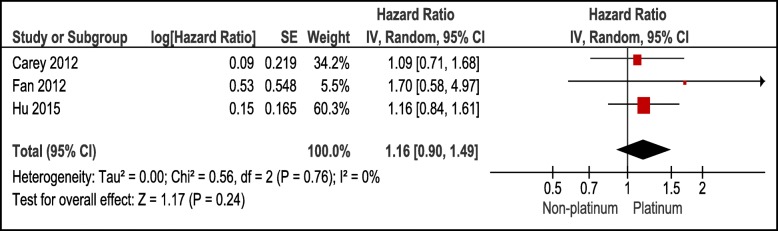
Forest plot showing pooled Hazard ratios in platinum vs non-platinum chemotherapy in metastatic TNBC using random effects model with 95% confidence interval

Three studies (*N* = 531) evaluated the PFS rates among TNBC patients. Figure 4 showed that the difference in PFS rates was not statistically significant (*P* = 0.24) among those treated with platinum-based regimen compared to those treated with non-platinum based regimen. Studies and results were homogenous with an I^2^ of 0.

**Fig. 5:**
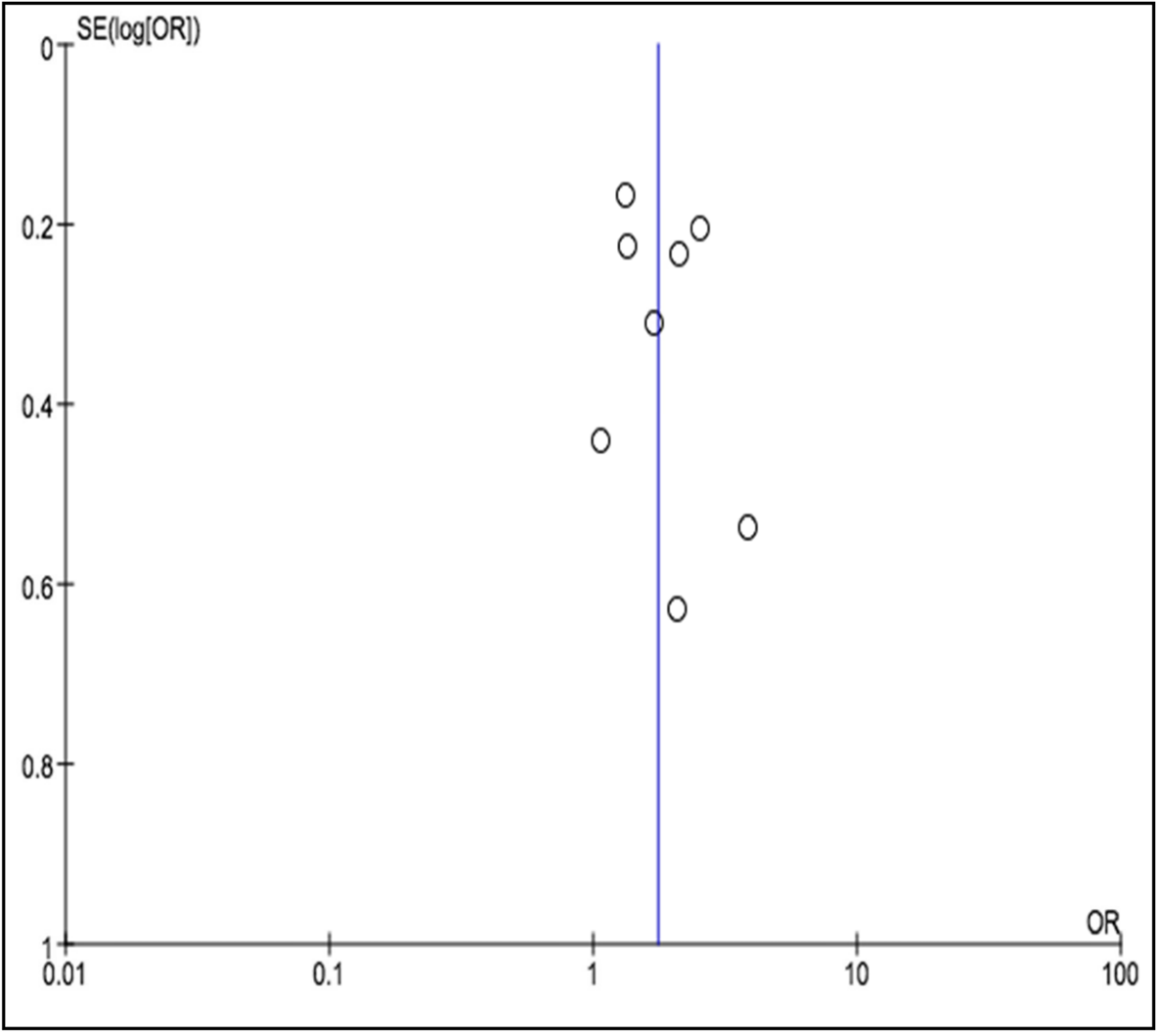
Funnel plot of the pCR rate in TNBC patients who were treated with a platinum-or non-platinum-based regimen

### Safety profile

In both neoadjuvant and metastatic settings, toxic effects such as anaemia, neutropenia, thrombocytopenia, and nausea, occurred more commonly in the group given platinum. The addition of platinum was also associated with a higher rate of diarrhoea and anorexia. On the other hand, skin rash, nail changes, pneumonitis, and other cardiac disorders were more common in the group not treated with platinum. Patients assigned to the platinum arm were more likely to require dose reduction or stop treatment early because of toxicity.

## Discussion

Despite progress, TNBC still has significantly lower responses to therapy compared to other molecular subtypes of breast cancer. Several factors hinder treatment of TNBC, such as: a high tendency to metastasize to other organ sites, lack of FDA-approved targeted therapies, high rates of recurrence after diagnosis, and extreme heterogeneity of TNBCs [2].

The response to neo-adjuvant chemotherapy varies with breast cancer molecular subtype. Studies have shown that both TNBC and Her2-positive breast cancer have excellent prognosis once pCR is achieved after neo-adjuvant chemotherapy compared to other molecular subtypes [9]. There are six subtypes of TNBC based on molecular sub-typing and gene expression studies. These include: basal-like-1, basal-like-2, immune-modulatory, mesenchymal, mesenchymal-like and a luminal androgen receptor subtype [3]. Among the subtypes, 75% are basal-like and these are most commonly associated with BRCA1 mutations. Platinum compounds are found to be especially useful in cancer cells with deficiencies in DNA repair such as those with BRCA gene mutations, due to the formation of platinum-DNA adducts. The TNT phase III trial randomized 376 patients with metastatic TNBC to docetaxel or carboplatin. In the BRCA mutation carriers (*n* = 29) response rates to carboplatin were 68% compared to 30% for docetaxel [23]. It is therefore warranted to investigate this relationship between BRCA mutation and chemo-sensitivity.

The benefit of neoadjuvant chemotherapy among TNBC shown in our results is consistent with current recommendations. Among the 2415 TNBC patients who underwent platinum-based neoadjuvant chemotherapy, this study showed statistically significant improvement in pCR rates compared to non-platinum-based treatment. However, this data is affected by moderate heterogeneity among the studies, which has been associated with the varied agents combined with the platinum therapy. It remains unclear how platinum should be incorporated and whether concomitant use of platinum could be used to substitute for anthracycline, taxane or an alkylator. Subgroup analyses of the neoadjuvant platinum-based regimen showed that platinum combined with taxane has statistically significant improved pCR. This is consistent with the BrightTNess trial [22] in 2018 which showed that combination of carboplatin and paclitaxel increased pCR rates.

Platinum-containing agents are not regarded as a standard for neoadjuvant therapy of TNBC, for several reasons. One reason is that given that the addition of platinum results in greater toxicity as seen in the previous studies, the clinical benefits of its use should be clear. It is also possible that the improvements in pCR rates may be a result of down staging of low-volume residual disease, which is not known to translate to lower recurrence rates. In addition, pCR may not be associated with improved outcomes in BRCA1/2 mutation carriers, suggesting the inconsistency of its prognostic effect.

In the metastatic setting, this metanalysis did not show any advantage in terms of PFS among TNBC patients. Platinum-based chemotherapy has been suggested to potentially be more effective than non-platinum-based chemotherapy in metastatic TNBC. In the CBCSG006 trial by Hu et al. [17], where Gemcitabine was used as the backbone, Cisplatin was compared to Paclitaxel as a first line metastatic treatment. Over-all response rate was higher in Cisplatin-Gemcitabine combination than in Paclitaxel-Gemcitabine (64% vs 49%, *p* < 0.018) with a PFS advantage of 1.26 months (HR 0.692, 95% CI 6.19–9.30, *p* < 0.0001). No overall survival difference was noted. However, several limitations were noted in this particular study including potential bias in the definition of TNBC and financial limitations which preclude central assessment, and further classification into TNBC subtypes.

The TBCRC 001 trial divided the study cohorts into three arms and investigated Cetuximab with or without Carboplatin. Expression of Epidermal Growth Factor Receptor (EGFR), a key gene in the basal-like TNBC, was assessed. However, the limited activity of Cetuximab shown in this study by Carey et al. [16]. suggests that TNBC may have constitutive pathway activation via downstream components such as KRAS amplification or CRYAB expression. The study by Fan et al. [25], with a Taxane-based treatment compared Cisplatin (TP) and Capecitabine (TX). Regardless of the metastasis, the response rates were noted to be higher in the TP arm than in the TX arm (63% vs 15.4%, *P* = 0.001). Median PFS and Median OS were also statistically longer in the TP arm.

The current conflicting data using platinum among TNBC patients in the metastatic setting may not be sufficient to warrant further study, however, the potential of platinum therapy in certain subtypes of TNBC may be explored further.

## Conclusion

Platinum-based systemic treatment is associated with statistically significant improved pCR rates among patients with TNBC in the neoadjuvant setting. Subgroup analysis of homogenous data further delineated that the combination of platinum and taxanes improved pCR rates in the same population. With these results, a platinum-taxane regimen may be a justifiable treatment regimen for operable TNBC. On the other hand, in the metastatic setting, platinum-based regimen did not show statistically significant advantage in PFS. BRCA1 mutation determination for all TNBC patients may cause a potential paradigm shift in the management of these type of patients in the future.

## Data Availability

All data generated or analysed during this study are included in this published article and referenced articles are listed in the References section.

## Abbreviations

CI: Confidence interval
DFS: Disease-free survival
DNA: Deoxyribonucleotide acid
HR: Hazard ratio
NCCN: National Comprehensive Cancer Network
OR: Odds ratio
ORR: Overall response rate
OS: Overall survival
pCR: Pathologic complete response
PFS: Progression-free survival
RCT: Randomized controlled trial
TNBC: Triple negative breast cancer
